# A Mutant Isoform of ObgE Causes Cell Death by Interfering with Cell Division

**DOI:** 10.3389/fmicb.2017.01193

**Published:** 2017-06-28

**Authors:** Liselot Dewachter, Natalie Verstraeten, Michiel Jennes, Tom Verbeelen, Jacob Biboy, Daniel Monteyne, David Pérez-Morga, Kevin J. Verstrepen, Waldemar Vollmer, Maarten Fauvart, Jan Michiels

**Affiliations:** ^1^Centre of Microbial and Plant Genetics, KU Leuven – University of LeuvenLeuven, Belgium; ^2^Centre for Bacterial Cell Biology, Institute for Cell and Molecular Biosciences, Newcastle UniversityNewcastle upon Tyne, United Kingdom; ^3^Laboratory of Molecular Parasitology, Institut de Biologie et de Médecine Moléculaires, Université Libre de BruxellesGosselies, Belgium; ^4^Center for Microscopy and Molecular Imaging, Université Libre de BruxellesGosselies, Belgium; ^5^Systems Biology Laboratory, VIB Center for MicrobiologyLeuven, Belgium; ^6^Department of Life Sciences and Imaging, Smart Electronics Unit, ImecLeuven, Belgium

**Keywords:** Obg, ObgE, cell division, cell cycle, cell cycle checkpoint, lysis, cell separation

## Abstract

Cell division is a vital part of the cell cycle that is fundamental to all life. Despite decades of intense investigation, this process is still incompletely understood. Previously, the essential GTPase ObgE, which plays a role in a myriad of basic cellular processes (such as initiation of DNA replication, chromosome segregation, and ribosome assembly), was proposed to act as a cell cycle checkpoint in *Escherichia coli* by licensing chromosome segregation. We here describe the effect of a mutant isoform of ObgE (ObgE^∗^) that causes cell death by irreversible arrest of the cell cycle at the stage of cell division. Notably, chromosome segregation is allowed to proceed normally in the presence of ObgE^∗^, after which cell division is blocked. Under conditions of rapid growth, ongoing cell cycles are completed before cell cycle arrest by ObgE^∗^ becomes effective. However, cell division defects caused by ObgE^∗^ then elicit lysis through formation of membrane blebs at aberrant division sites. Based on our results, and because ObgE was previously implicated in cell cycle regulation, we hypothesize that the mutation in ObgE^∗^ disrupts the normal role of ObgE in cell division. We discuss how ObgE^∗^ could reveal more about the intricate role of wild-type ObgE in division and cell cycle control. Moreover, since Obg is widely conserved and essential for viability, also in eukaryotes, our findings might be applicable to other organisms as well.

## Introduction

The bacterial cell cycle can be divided into three stages. The first stage, the B period, extends from cell birth until the initiation of DNA replication and is followed by the C period in which replication proceeds. Finally, after termination of replication, cell division occurs during the D period (Wang and Levin, 2009). Division starts with the formation of the proto-ring at future division sites (Lutkenhaus et al., 2012; Haeusser and Margolin, 2016). In *Escherichia coli*, the proto-ring consists of cytoplasmic FtsZ and its membrane tethers FtsA and ZipA, all of which are essential for cell division (Pichoff and Lutkenhaus, 2002, 2005; Adams and Errington, 2009). With the aid of FtsA and ZipA, the tubulin homolog FtsZ assembles into a dynamic ring-like polymer structure underneath the cytoplasmic membrane (Adams and Errington, 2009; Erickson et al., 2010; Lutkenhaus et al., 2012; Meier and Goley, 2014; Haeusser and Margolin, 2016). After a certain lag period, the proto-ring acts as a scaffold for the assembly of the divisome by recruiting the remaining cell division proteins to the division site (Chen and Beckwith, 2001; Aarsman et al., 2005; Lutkenhaus et al., 2012; Haeusser and Margolin, 2016). The precise function of many of these proteins remains unknown, but many of them appear to be involved in peptidoglycan metabolism. Recruitment of the final essential division protein, FtsN, triggers the activation of the divisome after which the septum is formed (Chen and Beckwith, 2001; Aarsman et al., 2005; Gerding et al., 2009). Septum formation is carried out by the divisome protein PBP3, a peptidoglycan transpeptidase also known as FtsI, in concert with at least one peptidoglycan transglycosylase, such as PBP1B (Bertsche et al., 2006; Typas et al., 2011; Lutkenhaus et al., 2012; Egan and Vollmer, 2013). During this process, invagination of both inner and outer membrane is coordinated with peptidoglycan synthesis to maintain a close association between these three cell envelope layers (Gray et al., 2015). Invagination of the inner membrane is driven by either peptidoglycan synthesis, Z-ring contraction or a combination of both (Meier and Goley, 2014), and treadmilling of FtsZ drives peptidoglycan synthesis (Bisson-Filho et al., 2017). Invagination of the outer membrane is promoted by the Tol-Pal system, a protein complex that may span the entire cell envelope. This complex uses proton motive force to aid in constriction and thus maintain a constant small distance between the outer membrane and the peptidoglycan layer (Cascales et al., 2001; Gerding et al., 2007). Moreover, the Tol-Pal system can interact with PBP1B through the periplasmic protein CpoB and modulate its activity, thus coupling outer membrane invagination with septal peptidoglycan synthesis (Gray et al., 2015). While constriction proceeds, the initially shared peptidoglycan layer of the septum is cleaved by the coordinated effort of several partially redundant peptidoglycan hydrolases. The peptidoglycan amidases (AmiA, AmiB, and AmiC) have the most prominent role in cell separation (Heidrich et al., 2002; Vollmer et al., 2008; Typas et al., 2011). Deletion of these amidases severely impedes the separation of daughter cells and results in the formation of long cell chains (Heidrich et al., 2001). Ultimately, after cytokinesis has completed and shared peptidoglycan is split, two separate daughter cells emerge.

Obg is a widely conserved GTPase that is essential for bacterial viability. By binding to GTP, GDP or ppGpp, the mediator of the stringent response, Obg can sense the cell’s energy status and act accordingly (Lin et al., 1999; Buglino et al., 2002; Wout et al., 2004; Persky et al., 2009). Obg depletion studies in *E. coli* have shown that when ObgE (Obg of *E. coli*) levels decline, cells become elongated and cease to divide (Kobayashi et al., 2001; Foti et al., 2007). Similar phenotypes are found upon overexpression of ObgE (Kobayashi et al., 2001; Dutkiewicz et al., 2002). Filamentation upon perturbation of ObgE levels was proposed to be caused by a defect in chromosome segregation (Kobayashi et al., 2001; Foti et al., 2007). This hypothesis is supported by the observation that aberrant chromosome segregation upon ObgE depletion is associated with inefficient FtsZ-ring formation (Foti et al., 2007). Apart from its effect on chromosome segregation, Obg plays a role in many other important cellular processes. For example, ObgE is involved in the initiation of DNA replication since a temperature-sensitive ObgE mutant failed to initiate replication at the non-permissive temperature. Under these conditions, the cellular concentration of the initiator protein DnaA is lowered, providing an explanation for the observed phenotype (Ulanowska et al., 2003; Sikora et al., 2006). Additionally, overexpression of ObgE results in overreplicated chromosomes and asynchronous initiation of replication, the latter of which was also detected upon ObgE depletion (Dutkiewicz et al., 2002; Foti et al., 2005, 2007). Depletion of ObgE, however, did not impede replication initiation and thus failed to reproduce the temperature-sensitive phenotype (Foti et al., 2007). Other processes in which ObgE is involved include replication fork stability, ribosome assembly, the stringent response and antibiotic tolerance (Wout et al., 2004; Foti et al., 2005; Jiang et al., 2006; Persky et al., 2009; Feng et al., 2014; Verstraeten et al., 2015). Based on its role in DNA metabolism, Obg was previously proposed to act as a cell cycle checkpoint capable of halting progression through the cell cycle and blocking cell division (Datta et al., 2004; Foti et al., 2005).

We previously identified ObgE_K268I_ (referred to as ObgE^∗^), a toxic isoform of the essential GTPase ObgE (Dewachter et al., 2015, 2016a). The K268I amino acid substitution is located in the G domain of ObgE which is responsible for nucleotide binding and hydrolysis. However, the K268 residue is not immediately involved in interactions with GTP or GDP (Gkekas et al., 2017). Since this residue is located at the surface of the protein, it might be involved in the interaction with effector molecules rather than influencing the nucleotide binding state of ObgE. When expressed in *E. coli*, ObgE^∗^ causes rapid cell death. Previous efforts to identify the pathway triggered by ObgE^∗^ have allowed us to exclude several known bacterial cell death pathways (Dewachter et al., 2015). However, the mechanism underlying ObgE^∗^-mediated cell death has remained elusive. We here show that ObgE^∗^ causes cell death by irreversibly halting the cell cycle through inhibition of cell division. Depending on conditions at the time of ObgE^∗^ expression, cell cycle arrest occurs instantaneously or is activated after one or two rounds of defective cell division in which the separation of daughter cells is prevented. Because of the previously proposed role of ObgE in the cell cycle (Kobayashi et al., 2001; Dutkiewicz et al., 2002; Foti et al., 2005, 2007), we postulate that wild-type ObgE is involved in the regulation of cell division and that this functionality is severely disturbed by the amino acid permutation present in ObgE^∗^. Investigation of the mechanism by which ObgE^∗^ interferes with these processes is therefore likely to reveal the role of ObgE in the regulation of the cell cycle and more specifically, its role in cell division.

## Results

### ObgE^∗^ Causes Loss of Viability and Lysis

**FIGURE 1:**
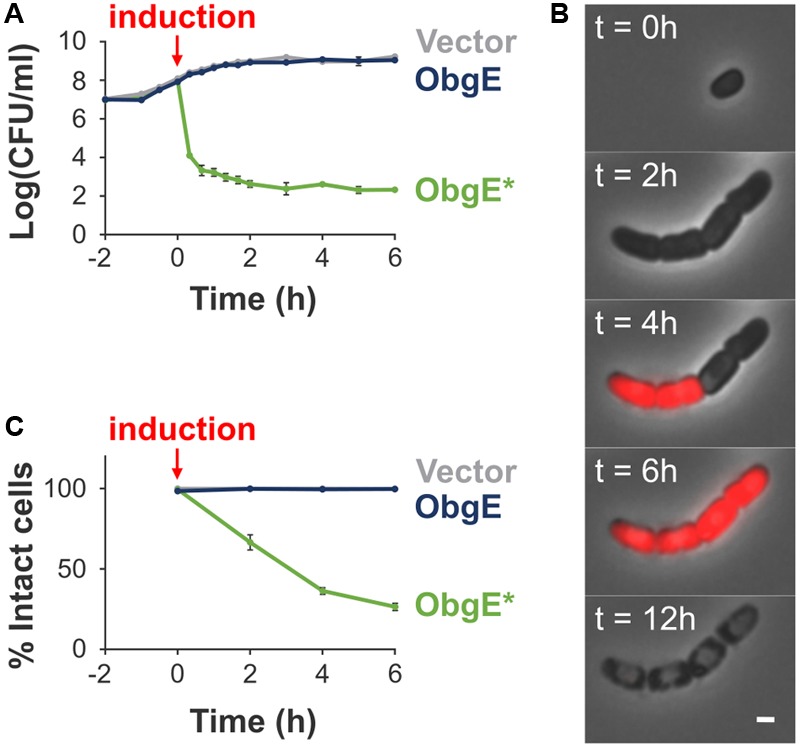
Characterization of ObgE^∗^-mediated cell death. **(A)** Exponential-phase cultures of *Escherichia coli* pBAD33, *E. coli* pBAD33-*obgE* or *E. coli* pBAD33-*obgE^∗^* were induced at time 0. At several time points before and after induction, the number of viable cells was determined by plate counting. Error bars represent the standard error of the mean, *n* ≥ 3. **(B)** Time lapse observations of *E. coli* pBAD33-*obgE^∗^* seeded on a lysogeny broth (LB) agar pad containing the inducer of ObgE^∗^ expression and propidium iodide (PI). Pictures were taken over a time course of 12 h. Scale bar, 1 μm. **(C)** Exponential-phase cultures of *E. coli* pBAD33, *E. coli* pBAD33-*obgE* or *E. coli* pBAD33-*obgE^∗^* were induced at time 0. At several time points after induction, cultures were stained with PI and the percentage of PI-negative and thus intact cells in the population was measured by flow cytometry. Data are represented as mean ± SEM, *n* ≥ 3. In every repeat 100,000 cells were collected.

We previously discovered a dominant-negative isoform of ObgE that causes cell death in *E. coli*. This mutant isoform contains a single amino acid substitution, K268I, and is called ObgE^∗^ (Dewachter et al., 2015, 2016a,b). When ObgE^∗^ is expressed, it causes a drastic reduction in the number of viable cells, as measured by colony forming units (CFUs). In contrast, expression of wild-type ObgE does not influence viability in comparison to the vector control (**Figure 1A**). The drop in viability caused by ObgE^∗^ is accompanied by a loss of membrane integrity, which can be detected by staining with propidium iodide (PI), a red fluorescent dye that can only enter cells with compromised membranes. To reconstruct in detail the order of events occurring upon ObgE^∗^ expression, we carried out time lapse experiments of *E. coli* expressing ObgE^∗^ in the presence of PI (**Figure 1B**). First, ObgE^∗^ very rapidly causes a defect in cell separation; newly formed daughter cells fail to separate and instead remain together in a cell chain. After one or two rounds of defective cell division, cells cease to grow and divide and start staining PI-positive, indicating that their membrane integrity is lost. Remarkably, not all cells in one chain stain PI-positive at the same time, indicating that at least in some cases constriction has proceeded normally and has separated the cytoplasm of the daughter cells. Cells that stain PI-positive are able to maintain this PI staining over several hours. However, over a time course of approximately 10–12 h, cytoplasmic content together with PI is lost from the cell, indicating that ObgE^∗^ causes stepwise, slowly progressing cell lysis. Since all PI-positive cells eventually lyse and PI-negative cells remain intact, we can quantify lysis by PI staining, as was done previously (Packard et al., 2013). Because individual cells in a chain were never able to remain intact when parts of the chain stained PI-positive, the entire chain was considered to be compromised if at least one cell lost its membrane integrity. This analysis shows that ObgE^∗^ triggers lysis in the majority of the population, while virtually all cells remain intact upon expression of wild-type ObgE (**Figure 1C**).

### Lysis Proceeds through Formation of Membrane Blebs

**FIGURE 2:**
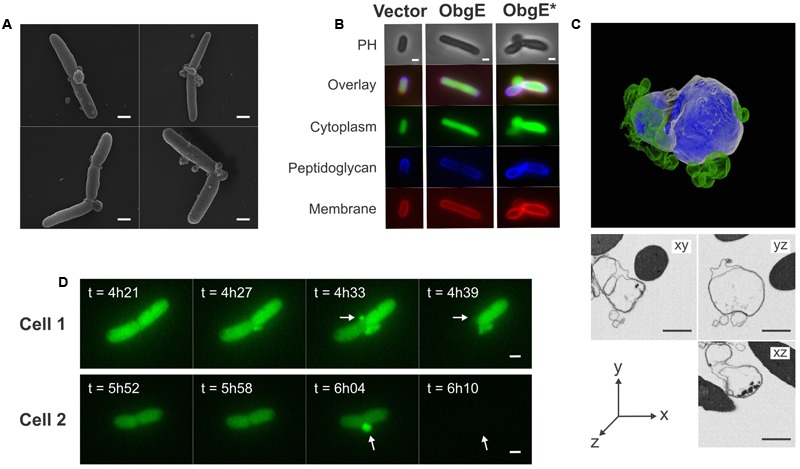
Lysis is caused by rupturing of membrane blebs that consist of both outer and inner membrane. **(A)** Scanning electron microscopy images of *E. coli* overexpressing ObgE^∗^ show the formation of membrane blebs. **(B)** Microscopy images of *E. coli* pBAD33, *E. coli* pBAD33-*obgE* or *E. coli* pBAD33-*obgE^∗^* with a cytoplasmic GFP label encoded by pQE80L-*gfp*. Cells were stained with HADA for visualization of peptidoglycan and FM4-64 for labeling of membranes. PH, phase contrast. **(C)** 3D-model of a bleb constructed by focused ion beam-scanning electron microscopy (FIB-SEM). The outer membrane is shown in white, inner membrane in blue and accessory blebs that are not in direct contact with the major bleb are shown in green. Additionally, sections from this bleb from three different angles are shown. **(D)** Time lapse images showing the formation of blebs and lysis of *E. coli* pBAD33-*obgE^∗^* pQE80L-*gfp* upon induction with arabinose. Blebs that cause lysis are indicated with arrows. Scale bars, 1 μm.

A detailed study of *E. coli* morphology by scanning electron microscopy revealed that ObgE^∗^ expression leads to the formation of membrane protrusions, termed blebs (**Figure 2A**). Similar membrane structures were previously associated with cell lysis (Yao et al., 2012; Sutterlin et al., 2016). The excess amount of membrane that forms blebs points to disturbance of membrane homeostasis by ObgE^∗^. To gain further structural insight into the nature of these blebs, the cytoplasm, membranes and peptidoglycan of *E. coli* expressing ObgE^∗^ were simultaneously labeled (**Figure 2B**). Cytoplasm was visualized by the expression of a cytoplasmic GFP label, membranes were stained with the red lipophilic dye FM4-64, and peptidoglycan was visualized using HADA [HCC-amino-D-alanine, a fluorescently labeled D-amino acid that is readily incorporated into the peptides of peptidoglycan (Kuru et al., 2015)]. No membrane blebs were found in the vector control or *E. coli* expressing wild-type ObgE, although the latter did influence cell morphology by increasing cell length, in accordance with literature (Kobayashi et al., 2001; Dutkiewicz et al., 2002). Expression of ObgE^∗^ leads to the formation of membrane blebs that contain the cytoplasmic GFP label. The lumen of these blebs is therefore in direct contact with the cytoplasm. Because of this continuum between cytoplasm and blebs, it is likely that they are lined by inner as well as outer membrane. The presence of inner membrane inside blebs was confirmed by construction of a 3D-image of blebs by focused ion beam-scanning electron microscopy (FIB-SEM), a technique that allows for high resolution imaging of a desired volume in three dimensions by electron microcopy (Kizilyaprak et al., 2014) (**Figure 2C**). However, although blebs contain inner membrane, there is no clear defect in the underlying peptidoglycan layer since HADA labeling is uniform and uninterrupted at the site of bleb formation. Any potential peptidoglycan defect allowing for the protrusion of inner membrane should therefore be rather small. Additionally, the composition of peptidoglycan remains unaltered in the presence of ObgE^∗^, arguing against major rearrangements or disturbance of peptidoglycan structure (Supplementary Figure S1). FM4-64 and HADA staining revealed that blebs are membrane structures that lack the rigidity and protection of the peptidoglycan layer. Membrane blebs are therefore highly fragile structures that are prone to rupturing (Yao et al., 2012). Indeed, blebs usually have short life spans and, importantly, their rupturing coincides with loss of the cytoplasmic GFP label not only from the lumen of the bleb but from the entire cell (**Figure 2D**). Taken together, these data indicate that lysis proceeds through the formation of membrane blebs that contain both outer and inner membrane and thus are connected to the cytoplasm. When these blebs rupture, cytoplasmic content is released and lysis occurs.

### Blebs Preferentially Form at Division Sites

**FIGURE 3:**
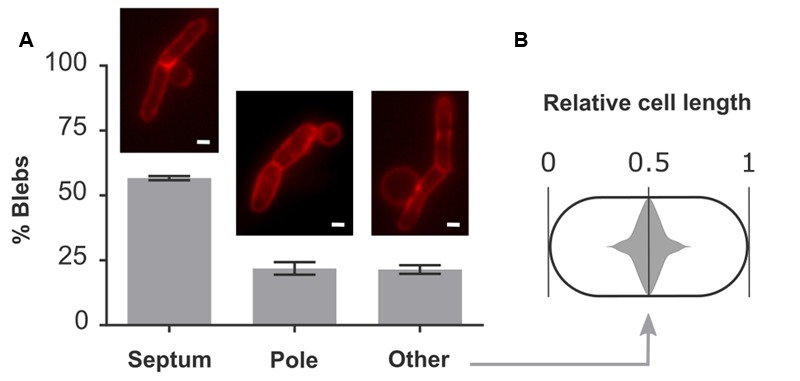
Blebs are located at sites of cell division. **(A)** Quantitative localization of blebs in *E. coli* pBAD33-*obgE^∗^*. Cultures were induced in the presence of 100 mM MgSO_4_ to increase bleb size and lifetime and thus improve visibility under the microscope. Membranes were stained with FM4-64 before visualization. Data are represented as mean ± SEM, *n* = 3. In every repeat ± 200 blebs were counted. **(B)** Violin plot of the distribution of blebs from the category ‘Other’ within the cell. To account for the random distribution of bleb formation in either the left hand or right hand side of the cell, all data points were duplicated and mirrored around midcell position. Scale bars, 1 μm.

Quantification of the localization of blebs showed that in 57% of all cases, blebs were situated on top of a fully formed septum (**Figure 3A**). These defective septa are not resolved to form two entirely separate and unlinked daughter cells but instead remain part of a cell chain. The predominant occurrence of blebs at these aberrant division sites indicates that an additional defect in these septa allows for the formation of blebs. 22% of blebs were found at cell poles and the remaining 21% were located elsewhere in the cell (**Figure 3A**). Interestingly, blebs belonging to the latter category are not randomly located along the cell length but instead are centered around midcell position (**Figure 3B**). Since cell division occurs at this location, these blebs likely arise through a division defect. Together with blebs that are formed on top of a septum, this makes for a total of 78% of blebs that are formed at division sites, either before or after constriction. Since cell lysis proceeds through the formation and rupture of blebs, this observation hints at an important role for cell division in the progression of lysis.

### Cell Division Is Essential for Lysis But Not for Loss of Viability

**FIGURE 4:**
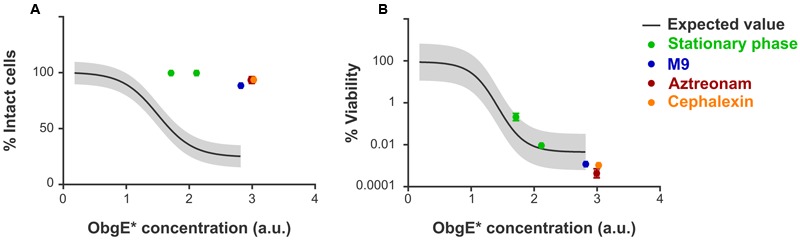
Cell division is necessary for lysis but does not affect loss of viability. **(A)** Correlation curve showing the expected fraction of intact cells of *E. coli* pBAD33-*obgE^∗^* in function of the intracellular ObgE^∗^ concentration. **(B)** Correlation curve showing the expected level of viability of *E. coli* pBAD33-*obgE^∗^* in comparison to *E. coli* pBAD33-*obgE* in function of the intracellular ObgE^∗^ concentration. Colored data points were collected from conditions that inhibit or slow down cell division. ObgE^∗^ concentration was determined by measuring fluorescence of an ObgE^∗^-Venus fusion by flow cytometry. Gray bands around the expected value represent 99% prediction intervals. Data are represented as mean ± SEM, *n* ≥ 3, error bars are mostly too small to be visible.

Since ObgE^∗^ causes cell chaining and thus clearly interferes with normal progression of cell division, and because the majority of blebs are found at division sites and thus likely occur through a septum defect, we investigated the role of cell division in the progression of lysis. To do this, several conditions were selected in which division is either inhibited or severely slowed down. First, ObgE^∗^ was expressed at two different time points in stationary phase and its effect on lysis and loss of viability was determined by PI staining and plate counting, respectively. Likewise, we examined the effect of ObgE^∗^ during growth in M9 minimal medium, where the doubling time of *E. coli* is approximately two times higher than in lysogeny broth (LB) and cells thus only divide half as fast (Supplementary Figure S2). However, since both conditions also have a large effect on general metabolism and other aspects of cell growth, the role of cell division was also investigated more directly by expressing ObgE^∗^ in the presence of either aztreonam or cephalexin, two antibiotics that specifically inhibit division. They do so by mainly targeting PBP3 and thus inhibit the synthesis of septal peptidoglycan while having no or very limited effects on cell elongation (Satta et al., 1995; Yao et al., 2012). It is likely that some of these conditions affect the expression level of ObgE^∗^ inside the cell. To account for this, lysis and loss of viability were first quantified under standard conditions at 25 different ObgE^∗^ concentrations. These data were used to construct a correlation curve that reflects the extent of lysis and loss of viability at every possible concentration of ObgE^∗^ within a certain range (Supplementary Figure S3). In conditions that slow down or inhibit cell division, the percentage of intact cells is greatly increased and is much higher than what is expected based on the expression level (**Figure 4A**). In fact, in all conditions where cell division is inhibited (i.e., ObgE^∗^ expression in stationary phase or in the presence of aztreonam or cephalexin), the percentage of intact cells approaches 100%, indicating that virtually no lysis occurs anymore. Cell division therefore appears to be essential for the progression of ObgE^∗^-mediated lysis, more specifically we postulate that a defect in septal peptidoglycan metabolism is responsible for lysis. When ObgE^∗^ is expressed in M9 minimal medium there is still a detectable amount of lysis. This is to be expected since cell division and septal peptidoglycan synthesis still occur in minimal medium, albeit with a lower frequency. To validate that it is division and/or septal peptidoglycan synthesis that causes lysis and not merely peptidoglycan synthesis in general, the amount of lysis was also measured in the presence of mecillinam. Mecillinam is an antibiotic that targets PBP2 and thus specifically inhibits peptidoglycan synthesis for cell elongation while having no effect on septal peptidoglycan synthesis (Satta et al., 1995). In the presence of this antibiotic, the percentage of intact cells is exactly what is expected based on the expression level, confirming that it is indeed cell division that is necessary for lysis to occur (Supplementary Figure S4).

In addition to estimating lysis by PI staining, ObgE^∗^-mediated toxicity was also quantified by CFU counting. Since the latter assay is based on cell growth, it determines the viability of cells instead of the integrity of the membrane as PI staining does. As shown in **Figure 4B**, expression of ObgE^∗^ in stationary phase, in M9 minimal medium or in the presence of either aztreonam or cephalexin has no effect on ObgE^∗^-mediated loss of viability. Measured values are located within the 99% prediction bands and thus do not significantly differ from the expected value. We therefore conclude that, while cell division is essential for the progression of lysis, it does not affect ObgE^∗^-mediated loss of viability.

### Lysis Is Not Caused by Amidases or the Tol-Pal System

**FIGURE 5:**
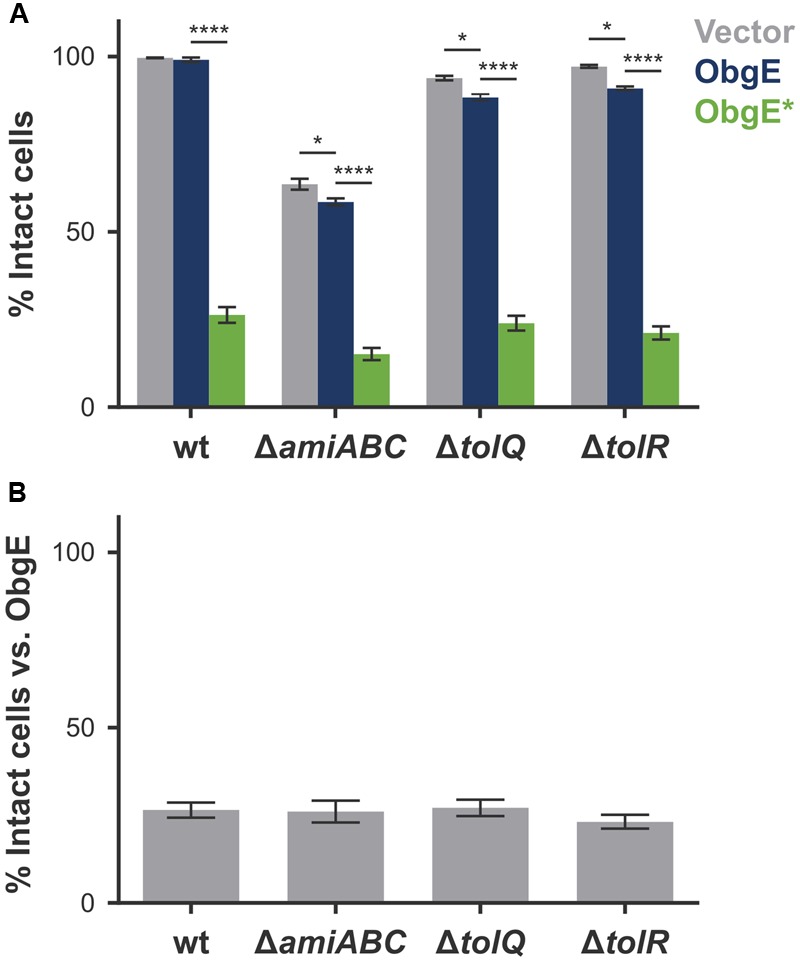
Amidases and the Tol-Pal system do not directly cause ObgE^∗^-mediated lysis. **(A)** Induced cultures of *E. coli* (wt), *E. coli* Δ*amiA* Δ*amiB* Δ*amiC* (Δ*amiABC*), *E. coli* Δ*tolQ* and *E. coli*Δ*tolR* with plasmids pBAD33, pBAD33-*obgE* or pBAD33-*obgE^∗^* were stained with PI and the percentage of PI-negative and thus intact cells in the population was measured by flow cytometry. **(B)** The percentage of intact cells upon ObgE^∗^ expression was divided by the fraction of intact cells upon expression of ObgE to correct for the differences in baseline levels of membrane integrity in different strains. Data are represented as mean ± SEM, *n* ≥ 3. In every repeat 100,000 cells were collected (one-way ANOVA, Bonferroni correction: ^∗^*p* < 0.05, ^∗∗∗∗^*p* < 0.0001).

The cell chaining phenotype caused by ObgE^∗^ points to a defect in the separation of newly formed daughter cells. Moreover, at these unresolved division sites, the cytoplasmic membrane is able to penetrate the peptidoglycan layer to form blebs that cause lysis. Both phenotypes could be caused by a faulty regulation of the amidases AmiA, AmiB, and AmiC. Although many enzymes cooperate to achieve cell separation, they are seen as the main executioners of this final stage of cell division (Heidrich et al., 2002; Vollmer et al., 2008; Typas et al., 2011). In their absence, *E. coli* forms chains of cells up to 20 cells long that are unable to separate (Heidrich et al., 2002). A lack of amidase activity thus causes cell chaining. Moreover, since amidases are peptidoglycan hydrolases, uncontrolled activity could also cause excessive degradation of peptidoglycan, leading to cell lysis. Another system of interest is the Tol-Pal protein complex that helps maintain a fixed distance between all three envelope layers (Gerding et al., 2007; Gray et al., 2015). Disruption of this system leads to a moderate cell chaining phenotype in medium of low osmolarity. Moreover, in the absence of a functional Tol-Pal complex, cells form outer membrane vesicles at division sites and cell poles (Gerding et al., 2007). Because of the obvious similarities with phenotypes associated with ObgE^∗^-mediated lysis, we quantified lysis in an *E. coli* triple amidase knock-out strain (*E. coli* Δ*amiA* Δ*amiB* Δ*amiC*) and in single-gene knock-outs of the *tolQ* or *tolR* genes (**Figures 5A,B**). In all three strains, membrane integrity is decreased under control conditions; whereas 99.6% of a wild-type population is intact, this value drops to 63.6% by deletion of *amiA, amiB*, and *amiC*, and is lowered to 93.8 and 97.1% in the Δ*tolQ* and Δ*tolR* strain, respectively (**Figure 5A**, Vector). Surprisingly, overexpression of the essential ObgE protein further decreases membrane integrity of these knock-out strains. Upon expression of ObgE, integrity decreases by 5.1% in the triple amidase knock-out strain (*p* = 0.0322), 5.7% in the Δ*tolQ* strain (*p* = 0.0471), and 6.2% in the Δ*tolR* strain (*p* = 0.0305). Since ObgE has no effect on the integrity of a wild-type strain (*p* = 0.999), these remarkable results hint at a functional link between ObgE, the amidases and the Tol-Pal complex.

ObgE^∗^ expression still has a very strong negative effect on cell integrity in all three deletion mutants. The amount of ObgE^∗^-mediated lysis varies slightly in the different strains, owing to the fact that the baseline of membrane integrity is affected by the genomic deletions. When the amount of lysis caused by ObgE^∗^ is normalized to the amount of lysis detected when wild-type ObgE is expressed, no significant differences in the percentage of intact cells were found among the three deletion strains (**Figure 5B**). Since lysis upon ObgE^∗^ expression does not decrease in the absence of AmiA, AmiB, and AmiC or in the absence of a functional Tol-Pal complex, we conclude that none of these proteins directly cause ObgE^∗^-mediated lysis.

### ObgE^∗^ Causes Loss of Viability by Irreversible Inhibition of the Cell Cycle

**FIGURE 6:**
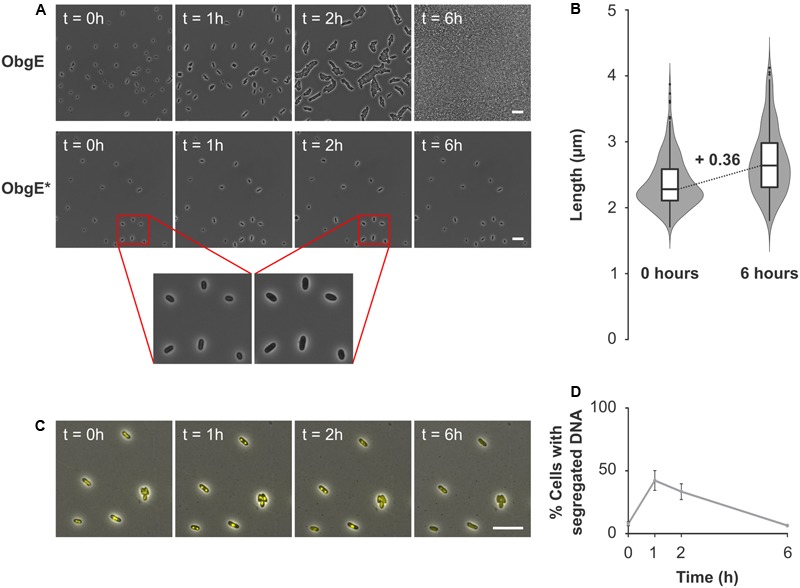
ObgE^∗^ causes irreversible cell cycle arrest. **(A)** After ObgE or ObgE^∗^ was expressed in stationary phase, cells were seeded on an agarose pad without the inducer of expression. Resumption of growth on the pad was monitored by time lapse microscopy. Insets are enlargements indicated by red lines. **(B)** Violin plot and box plot of the cell length of *E. coli* pBAD33-*obgE^∗^* at the start of the time lapse experiment and 6 h later. Cell length was quantified in ±220 cells spread over three independent repeats. **(C)** After ObgE^∗^ was expressed in *E. coli hupA-venus* in stationary phase, cells were seeded on an agarose pad without the inducer of expression. Chromosome segregation was monitored by time lapse microscopy. **(D)** Quantitative analysis of chromosome segregation in *E. coli hupA-venus* pBAD33Gm-*obgE^∗^* after induction in stationary phase. Data are represented as mean ± SEM, *n* = 4. In every repeat ± 100 cells were analyzed. Scale bars, 10 μm.

When lysis is prevented, ObgE^∗^ expression still decreases cell viability (**Figure 4**). To determine how ObgE^∗^ does so, the resumption of growth after ObgE^∗^ expression was monitored by time lapse microscopy. To separate the lysis and loss of viability phenotypes, ObgE^∗^ was expressed in stationary phase where lysis is prevented. Afterward, cells were seeded on an LB agarose pad lacking the inducer of ObgE^∗^ expression and incubated to allow cell growth. These conditions thus mimic the plate counting experiments in which a loss of viability is detected. In the control experiment where wild-type ObgE was expressed during stationary phase, cells rapidly resume growth after they are transferred to fresh medium. They quickly start to divide and form colonies (**Figure 6A**). When ObgE^∗^ was expressed during stationary phase, plate counting experiments demonstrated that there is a drop of 6 log units in colony formation (**Figure 1A**). This time lapse experiment showed that colonies fail to develop because cell division cannot be restored when cells are allowed to recover on fresh medium (**Figure 6A**). Even after 48 h, cells have not divided (data not shown). However, bacteria do appear to resume cell growth briefly (**Figure 6A**, inset). Quantification of cell length at the start of the time lapse and 6 h later revealed that, during this time, cell length increases to a limited extent (**Figure 6B**). ObgE^∗^ thus causes loss of viability by blocking progression through the cell cycle after allowing a limited amount of cell elongation but before cell division occurs. Importantly, this cell cycle arrest is not specific to exit from stationary phase and/or elevated (p)ppGpp levels. When ObgE^∗^ was expressed in exponential phase in the presence of aztreonam to prevent cell lysis, cell division could not be restored once cells were washed and resuspended in fresh medium (Supplementary Figure S5). Eventually, the arrested cells lose membrane integrity, as evidenced by gradually progressive PI staining. No membrane blebs were observed (data not shown).

Because of the role of ObgE in licensing chromosome segregation and subsequent cell division (Kobayashi et al., 2001; Foti et al., 2007), we checked whether ObgE^∗^ causes cell cycle arrest at the stage of chromosome segregation. To this end, the distribution of DNA inside the cell was monitored by a fluorescent fusion of *venus* to the *hupA* subunit of the general nucleoid-associated HU protein. Results indicate that chromosome segregation is not prevented by ObgE^∗^ (**Figure 6C**). One hour after transfer from stationary phase to fresh medium, chromosome segregation has occurred in approximately 42% of all cells. After this point, segregated nucleoids slowly start to reunite and spread throughout the cell (**Figure 6D**). However, even cells with segregated nucleoids fail to divide. This experiment demonstrates that it is not a defect in chromosome segregation *per se* that inhibits further progression through the cell cycle. We thus conclude that ObgE^∗^ interferes with another stage of the cell cycle. This point of interference is located after completion of chromosome segregation and inhibits the onset of cell division.

## Discussion

In this study, we describe the effect of a dominant-negative mutation in the essential GTPase ObgE on *E. coli*. Expression of the mutant ObgE^∗^ protein halts the cell cycle. This cell cycle arrest occurs after completion of chromosome segregation but before the onset of constriction for cell division. It is irreversible and therefore leads to cell death. Under conditions of rapid cell division at the time of ObgE^∗^ expression, cell death is associated with cell chain formation and lysis.

Since lysis can be prevented without affecting the loss of viability caused by ObgE^∗^, it is not a prerequisite for ObgE^∗^-mediated cell death. The underlying cause of cell death rather stems from ObgE^∗^’s capability to irreversibly halt the cell cycle and thus ultimately prevent cell division and colony formation. However, it is currently unclear at which point ObgE^∗^ halts the cell cycle to prevent the formation of daughter cells. Results indicate that ObgE^∗^ does not merely inhibit chromosome segregation, since a considerable fraction of the population displayed separated nucleoids without initiating cell division. Previously, filamentation by either depletion or overexpression of ObgE was suggested to be coupled to a defect in chromosome segregation (Kobayashi et al., 2001; Foti et al., 2007). However, our data show that cell division is prevented even when chromosomes successfully segregate, suggesting that the observed ObgE-related phenotypes, filamentation and aberrant chromosome segregation, might not be as tightly linked as previously thought. Neither does ObgE^∗^ inhibit cell growth in general, since limited elongation could be observed after ObgE^∗^ expression. However, it also seems unlikely that ObgE^∗^ inhibits cell division by directly affecting divisome activity, since the amount of elongation that occurs is not consistent with this possibility. When cell growth is halted by ObgE^∗^, median cell length has increased by 0.36 μm, which is only ±16% of the median cell length. At this point, a mature divisome most likely has not assembled since cell size approximately doubles before constriction occurs (Chien et al., 2012). Moreover, specific inhibition of divisome activity, for example by treatment with aztreonam or cephalexin, allows for more extensive cell elongation resulting in filaments that are much longer than the ones formed by cells that have expressed ObgE^∗^ (Vollmer and Bertsche, 2008; Yao et al., 2012). At which point does ObgE^∗^ then block the cell cycle? Interference must occur between the completion of chromosome segregation and the onset of constriction. A vital process that occurs during this time is the formation of the proto-ring, and thus the localization of FtsZ, at midcell. Interestingly, FtsZ localization was previously shown to be influenced by ObgE (Foti et al., 2007). Upon recruitment of FtsZ to the site of cell division, the mode of peptidoglycan growth switches from dispersed growth to preseptal elongation (Aarsman et al., 2005; Aaron et al., 2007; Vollmer and Bertsche, 2008; Olrichs et al., 2011; Typas et al., 2011). During the former, cells elongate through insertion of peptidoglycan precursors along the sidewalls. This insertion is directed by MreB and mediated by PBP1A and PBP2 (Vollmer and Bertsche, 2008; Typas et al., 2011). When FtsZ and ZipA localize at midcell, however, insertion of new peptidoglycan is redirected to midcell position (de Pedro et al., 1997; Aaron et al., 2007; Olrichs et al., 2011; Potluri et al., 2012). This preseptal peptidoglycan synthesis, also known as PIPS (PBP3-independent peptidoglycan synthesis), forms the transition between elongation and division and is independent of MreB, PBP1A and other elongation-related proteins (Potluri et al., 2012). Since elongation can occur in the presence of ObgE^∗^, although much more limited than expected, it is possible that ObgE^∗^ interferes with only one of both independent elongation modes while allowing the other to proceed normally. Because of the FtsZ dependency of preseptal growth and the influence of ObgE on FtsZ localization (de Pedro et al., 1997; Foti et al., 2007; Olrichs et al., 2011; Potluri et al., 2012), it appears more likely that ObgE^∗^ interferes with this phase of cell elongation. However, it should be noted that although preseptal elongation greatly contributes to cell elongation in *Caulobacter crescentus*, it only has a limited effect on *E. coli* cell length (de Pedro et al., 1997; Aaron et al., 2007; Olrichs et al., 2011; Potluri et al., 2012). Regardless of which step is affected, it is clear that cell division is ultimately inhibited. This inhibition is irreversible and can consequently explain the loss of viability observed in the presence of ObgE^∗^.

Whether or not ObgE^∗^-mediated cell cycle arrest is associated with cell chain formation and lysis, depends on growth conditions at the time of ObgE^∗^ expression. When ObgE^∗^ is expressed during exponential-phase growth in rich medium, cell chaining occurs through a defect in cell separation. Usually, these cell chains consist of 2–4 cells (data not shown), indicating that 1 or 2 rounds of defective cell division have occurred before complete cell cycle arrest by ObgE^∗^ is activated. Why do these exponential-phase cells not immediately halt their cell cycle? We hypothesize that, depending on the stage of the cell cycle at the time of ObgE^∗^ expression, cells may already be committed to executing cell division even in the presence of the cell cycle inhibitor ObgE^∗^. Although no research has been done as to the nature of this decision point in the cell cycle, initiation of DNA replication is an attractive candidate. Not only is ObgE involved in the regulation of replication initiation (Ulanowska et al., 2003; Foti et al., 2005; Sikora et al., 2006), but the number of cells found in cell chains also corresponds to one or two rounds of replication being active at the time of ObgE^∗^ expression, which is the case in exponential-phase cells grown in rich medium (Foti et al., 2005; Nielsen et al., 2007). In eukaryotic cells, such early commitment to cell division indeed exists and is referred to as the restriction point. If, at the restriction point, conditions are favorable, eukaryotic cells will initiate a new cell cycle, proceed to S phase and replicate their genomes. These cells are subsequently obliged to divide even if favorable conditions come to an end (Rhind and Russell, 2012).

The lethal commitment to cell division during cell cycle arrest imposed by ObgE^∗^ results in cell lysis. Lysis proceeds through the formation and rupturing of membrane blebs at division sites, implicating the presence of excess membrane upon ObgE^∗^ expression. These membrane structures are filled with cytoplasmic content and are lined by both outer and inner membrane. The inner membrane therefore is able to penetrate the peptidoglycan layer, hinting at a defect in this part of the cell envelope. ObgE^∗^ thus appears to lead to peptidoglycan defects and excess membrane. Interestingly, both conditions can induce the transition from rod-shaped bacteria to L-forms, wall-less bacteria able to proliferate independently of the FtsZ-based division machinery (Mercier et al., 2013, 2014). The nature of the peptidoglycan defect caused by ObgE^∗^ is currently unknown. Fluorescent HADA labeling did not reveal an apparent breach in peptidoglycan at the site of bleb formation. Moreover, peptidoglycan composition remains unaltered in the presence of ObgE^∗^. These data argue against large-scale peptidoglycan degradation but cannot exclude small and localized effects. The possibility of ObgE^∗^ causing localized peptidoglycan defects is corroborated by the fact that blebs do not form randomly, but instead are preferentially localized at sites of cell division. Conclusive evidence demonstrating the tight link between cell division and formation of blebs that cause lysis was provided by monitoring lysis in the absence of cell division. Under these conditions, lysis is prevented completely. Even when division is blocked after divisome assembly by addition of the FtsI-inhibitors, aztreonam or cephalexin, lysis does not occur. We can therefore trace back the timing of bleb formation and lysis to the third and final stage of cell division, which consists of active constriction, septal peptidoglycan synthesis and cell separation. Overall, our results indicate that cells that are committed to executing cell division in the presence of ObgE^∗^ experience a defect in correct peptidoglycan metabolism during cell constriction. The integrity of the septum is thereby disrupted, causing bleb formation and subsequent lysis. As a side note, blocking cell division rescues all cells from undergoing cell lysis, while approximately 80% of blebs are found at division sites. Since the remaining 20% of blebs is localized at cell poles, we speculate that these are new poles that were formed very early after induction of ObgE^∗^ expression. At this time, cells are presumably still able to separate, although a peptidoglycan defect that causes bleb formation has already occurred during septum formation. The newly formed cell poles are therefore fragile and will allow bleb formation and subsequent lysis.

In search of systems that function during division and that can potentially contribute to cell lysis, we turned to the amidases AmiA, AmiB, and AmiC and the Tol-Pal system by investigating lysis in selected deletion mutants. Intriguingly, for the first time, a link was observed between wild-type ObgE and the amidases and between wild-type ObgE and the Tol-Pal system. In the absence of amidases or upon disturbance of the Tol-Pal complex, cells become sensitive toward ObgE expression and lose their membrane integrity. Apart from the previously published effect on FtsZ (Foti et al., 2007), we thus report two additional links between ObgE and the division machinery. However, the amount of lysis upon ObgE^∗^ expression is not altered in the selected deletion strains, indicating that ObgE^∗^ does not cause lysis by disturbing the link that exists between wild-type ObgE and amidases or Tol-Pal. This also means that although AmiA, AmiB, and AmiC and the Tol-Pal system are attractive candidates to mediate lysis during cell division, they are not the main executioners of lysis caused by ObgE^∗^.

In conclusion, we here present evidence for the involvement of ObgE in the regulation of the *E. coli* cell cycle, more specifically at the stage of cell division. Apart from the direct link we found between ObgE and the amidases AmiA, AmiB, and AmiC and between ObgE and the Tol-Pal complex, we additionally observed irreversible cell cycle arrest upon expression of a mutant ObgE isoform. Cell cycle arrest occurs after completion of chromosome segregation but before the onset of constriction and thus likely affects an early event in the preparation for cell division. This phenotype highly resembles that of an Era mutant (Britton et al., 1998). Like Obg, Era is an essential and widely conserved GTPase, which was suggested to act as a growth-rate regulated checkpoint of the *E. coli* cell cycle (Britton et al., 1998). Likewise, it was previously suggested that wild-type ObgE serves as a bacterial cell cycle checkpoint that can block or allow further progression through the cell cycle based on its nucleotide occupancy (Datta et al., 2004; Foti et al., 2005; Dewachter et al., 2016b). Because of the central position of ObgE in cellular metabolism and its previously observed effect on cell division, ObgE is an attractive candidate to monitor several different cellular processes and adjust the progression of the cell cycle accordingly. The presence of the mutation in ObgE^∗^ blocks the cell cycle indefinitely, regardless of any cellular input, and thus appears to disturb the function of wild-type ObgE in this process. If true, this mutant can be used as a valuable tool to gain more insight into the bacterial cell cycle and its regulation. Importantly, since Obg is widely conserved, including in eukaryotes, our findings might be applicable to other organisms as well.

## Materials and Methods

### Strains, Plasmids and Growth Conditions

Experiments were performed with *E. coli* K-12 BW25113, except for data shown in Supplementary Figure S3. Single-gene knock-out strains were obtained from the Keio collection (Baba et al., 2006). The *E. coli* BW25113 Δ*amiABC* strain was constructed by sequentially replacing the *amiA, amiB*, and *amiC* genes with a kanamycin resistance cassette by phage transduction from HSC085, HSC078, and HSC071, respectively (Chung et al., 2009). After replacement of the *amiA* and *amiB* genes, the resistance cassette was removed by FLP-mediated recombination prior to the subsequent round of gene deletion. Knock-out of the correct genes was confirmed by PCR. *E. coli* BW25113 *hupA*-*venus* was constructed by phage transduction starting from *E. coli* MG1655 *hupA-venus-Cm^R^* (Abram Aertsen, KU Leuven, personal communication). The chloramphenicol resistance cassette was excised by FLP-mediated recombination.

For all tests, unless indicated otherwise, overnight cultures were diluted 100 times in LB containing the appropriate antibiotics (ampicillin 100 μg/ml, chloramphenicol 35 μg/ml, gentamicin 25 μg/ml, and kanamycin 40 μg/ml) and incubated at 37°C with continuous shaking at 200 rpm. When the OD_595__nm_ reached 0.4, expression from pBAD/His A (**Figure 2A**), pBAD33Gm (**Figures 6C,D**, for construction see supplemental experimental procedures) or pBAD33 (all other figures) was induced with arabinose (0.2% w/v). Expression of GFP from pQE80L-*gfp* (Orman and Brynildsen, 2015) was induced by IPTG (1 mM) at the same time. An alternative protocol was followed to investigate the effect of cell division on toxicity; induction was either postponed until one of two possible points in stationary phase (10 or 16 h after dilution of the overnight culture), carried out in M9 medium with casamino acids (1% w/v) as carbon source, or simultaneously aztreonam (0.2 μg/ml), cephalexin (50 μg/ml) or mecillinam (0.64 μg/ml) were added to the cultures. The concentration of these antibiotics is situated between the minimal inhibitory concentration (MIC) and minimal bactericidal concentration (MBC) (Supplementary Table S1), so as to inhibit cell growth without causing cell death.

### Cell Viability Assay

Serial dilutions were prepared in 10 mM MgSO_4_ and plated on medium containing 1.5 % agar. After overnight incubation at 37°C, CFUs were determined and, if needed, the percentage viability was calculated by dividing the number of CFUs per ml obtained after ObgE^∗^ expression by the number of CFUs per ml after ObgE expression.

### Lysis Assay

At indicated time points or – when no time point was specified – 6 h after induction with arabinose, cultures were diluted 10 (exponential-phase cultures) or 100 (stationary-phase cultures) times in PBS containing 0.03 μM PI and incubated in the dark at room temperature for 15 min. The fraction of the population that stained PI-positive was measured by flow cytometry with a BD Influx cell sorter equipped with 488 and 561 nm lasers and standard filter sets. Flow cytometry experiments were repeated n times. In each repeat, 100,000 cells were collected.

### ObgE^∗^ Concentration

To determine the intracellular ObgE^∗^ concentration in several conditions, a translational ObgE^∗^-Venus fusion (Dewachter et al., 2016a) was expressed and Venus fluorescence was measured by flow cytometry. Presence of the Venus tag did not influence toxicity in any way (data not shown).

### Microscopy

To simultaneously visualize blebs, cytoplasm, membranes and peptidoglycan (**Figure 2B**), 0.5 mM HADA was added to the cultures at the time of induction with arabinose. At this time GFP expression was also induced by adding 1 mM IPTG. Two hours later, cultures were washed twice with PBS to remove unincorporated HADA and pellets were dissolved in LB medium containing the appropriate antibiotics, 0.2% arabinose, 1 mM IPTG, and 100 mM MgSO_4_ to stabilize blebs. Cultures were incubated for another 2 h to allow bleb formation and were then stained with 10 μg/ml FM4-64. After 10 min incubation in the dark at room temperature cells were visualized on a poly-L-lysine coated glass slide.

Visualization of membrane blebs in *E. coli* pBAD33-*obgE^∗^* was performed by adding 100 mM MgSO_4_ at the time of induction and staining cells with 10 μg/ml FM4-64 4 h later (**Figure 3A**). MgSO_4_ was added to stabilize blebs and thus increase their lifetime, as was done previously (Yao et al., 2012).

For the time lapse microscopy experiment showing PI staining (**Figure 1B**), an overnight culture of *E. coli* pBAD33-*obgE^∗^* was seeded on an LB agarose pad (2% w/v) containing the appropriate antibiotic, 0.2% arabinose and 0.06 μM PI. Cells were incubated at 37°C and growth was monitored for 12 h.

The time lapse microscopy experiment with a cytoplasmic GFP label (**Figure 2D**) was performed with *E. coli* pBAD33-*obgE^∗^* pQE80L-*gfp*. An overnight culture of this strain was seeded on an LB agarose pad containing the appropriate antibiotics, 0.2% arabinose and 1 mM IPTG. Cells were incubated at 37°C and growth was monitored for 12 h.

Resumption of growth and chromosome segregation after ObgE or ObgE^∗^ expression in stationary phase (**Figures 6A,C**) was monitored in a time lapse experiment where overnight cultures of *E. coli* pBAD33-*obgE* and *E. coli* pBAD33-*obgE^∗^* or *E. coli hupA-venus* pBAD33Gm-*obgE^∗^* were diluted and grown for 16 h into stationary phase. Expression was then induced by adding 0.2% arabinose for 2 h after which cells were seeded on LB agarose pads containing the appropriate antibiotics without arabinose. Cells were incubated at 37°C and growth was monitored for 12–48 h. Cell length or segregation of fluorescent foci were quantified by MicrobeJ (Ducret et al., 2016).

For all microscopy experiments, cells were imaged by a Nikon Ti-E inverted microscope with Qi2 CMOS camera and temperature controlled cage incubator.

### Scanning Electron Microscopy

Scanning electron microscopy of *E. coli* WM2949 Δ*recA* pBAD/His A-*obgE^∗^* was performed as described previously (Dewachter et al., 2015).

### Focused Ion Beam-Scanning Electron Microscopy (FIB-SEM)

Focused ion beam-scanning electron microscopy was performed as described previously (Vanwalleghem et al., 2015).

### Statistical Analysis

All statistical analyses were performed with GraphPad Prism 6. Normality of representative data from CFU counts (*n* = 30) and PI staining (*n* = 15) was verified by D’Agostino and Pearson omnibus normality test. The different samples were compared using one-way ANOVA with *p*-values obtained from Bonferroni’s multiple comparisons test.

## Author Contributions

Conceptualization, LD, NV, DP-M, WV, MF, and JM; Methodology, LD, NV, MF, and JM; Formal analysis, LD; Investigation, LD, MJ, TV, JB, DM; Writing – original draft, LD; Writing – Review and Editing, LD, NV, DP-M, KV, WV, MF, and JM; Visualization, LD; Supervision, NV, MF, and JM.

## Conflict of Interest Statement

The authors declare that the research was conducted in the absence of any commercial or financial relationships that could be construed as a potential conflict of interest.
