# Supplementary material for: A Mutant Isoform of ObgE Causes Cell Death by Interfering with Cell Division
Source: Front Microbiol. 2017 Jun 28;8:1193. doi: 10.3389/fmicb.2017.01193 (PMC5487468; doi:10.3389/fmicb.2017.01193)
Supplement: Supplementary file 1 [file Data_Sheet_1.DOCX]

Supplementary Material

**A Mutant Isoform of ObgE Causes Cell Death by Interfering with Cell Division**

Liselot Dewachter, Natalie Verstraeten, Michiel Jennes, Tom Verbeelen, Jacob Biboy, Daniel Monteyne, David Pérez-Morga, Kevin J. Verstrepen, Waldemar Vollmer, Maarten Fauvart, Jan Michiels^*^

*** Correspondence:** Corresponding Author: [jan.michiels@kuleuven.be](mailto:jan.michiels@kuleuven.be)

# Supplementary materials and methods

## Plasmid construction

pBAD33Gm constructs were made by replacing the *cat* gene of pBAD33 ([Guzman et al., 1995](#_ENREF_4)) with the *gtmR* gene from pJQ200 ([Quandt and Hynes, 1993](#_ENREF_7)) by Gibson assembly. The vector was amplified with primers tttagcttccttagctcc and tttttttaaggcagttattggtg, while the Gm-resistance cassette was amplified by primers ttaagggcaccaataactgccttaaaaaaagttaggtggcggtacttg and tcgagattttcaggagctaaggaagctaaagatgttacgcagcagcaac. PCR products were purified and incubated with the Gibson master mix (New England BioLabs) which contains three different enzymes; an exonuclease to create single-stranded 3’ overhangs, a polymerase to fill in gaps after annealing of complementary overhangs of vector and insert to each other, and a ligase to seal nicks in the DNA. After incubation at 50°C for 15 min, the DNA mix was transformed to *E. coli* BW25113. Transformants were selected on plates containing gentamicin and replacement of the resistance cassette was confirmed by Sanger sequencing.

## Peptidoglycan composition

*E. coli* BW25113 Δ*recA* cells containing plasmid pBAD33Gm, pBAD33Gm-*obgE* or pBAD33Gm-*obgE** were grown to an OD_595 nm_ of 0.4, at which point 0.2 % arabinose was added and the cells were grown for 1 hour. Peptidoglycan isolation and analysis of muropeptide composition followed published procedures ([Glauner, 1988](#_ENREF_3); [Bui et al., 2009](#_ENREF_1)). Briefly, cells were harvested by centrifugation at 4°C and pellets were resuspended in ice-cold PBS. This cell suspension was then added to a boiling 8 % SDS solution and boiled for 30 min. Next, the peptidoglycan was purified and digested with cellosyl. The resulting muropeptides were reduced with sodium borohydride and separated by high-pressure liquid chromatography.

## Doubling time

The OD_600 nm_ of an overnight culture of *E. coli* pBAD33 was adjusted to 0.3 in 10 mM MgSO_4_ and then diluted 60 times in either LB or M9 medium containing the appropriate antibiotic. Cells were incubated and the OD_600 nm_ was measured every 10 minutes during 24 hours by an automated Bioscreen C system (Bioscreen C MBR, Oy Growth Curves AB Ltd.). Log_10_-transformed OD_600 nm_ data were fitted to a Gompertz equation to extract the specific growth rate, which is the inverse of the doubling time ([Zwietering et al., 1990](#_ENREF_8)).

## Correlation curve

The fraction of intact cells in the population and viability were determined at 25 different ObgE* concentrations. ObgE* concentration was measured as described in the materials and methods section. The measured concentration values were Log_10_ transformed and fitted to a sigmoidal standard curve. Different ObgE* levels were achieved by adding different amounts of the inducer, arabinose, to *E. coli* WM2949 ([Morgan-Kiss et al., 2002](#_ENREF_6)) pJAT8-*araE* ([Khlebnikov et al., 2001](#_ENREF_5)). In this strain, the chromosomal *araE* and *araFGH* genes are deleted and *araE* expression is controlled by a constitutive promoter on the pJAT8 plasmid, thereby transforming the usual on-off response of the P*_BAD_* promoter to a homogeneous response at single-cell level ([Khlebnikov et al., 2001](#_ENREF_5); [Morgan-Kiss et al., 2002](#_ENREF_6)).

## Determination of minimal inhibitory concentration and minimal bactericidal concentration

MIC and MBC values were determined in LB medium according to standard protocols ([Gerits et al., 2017](#_ENREF_2)).

# Supplementary Figures and Tables

## Supplementary Figures


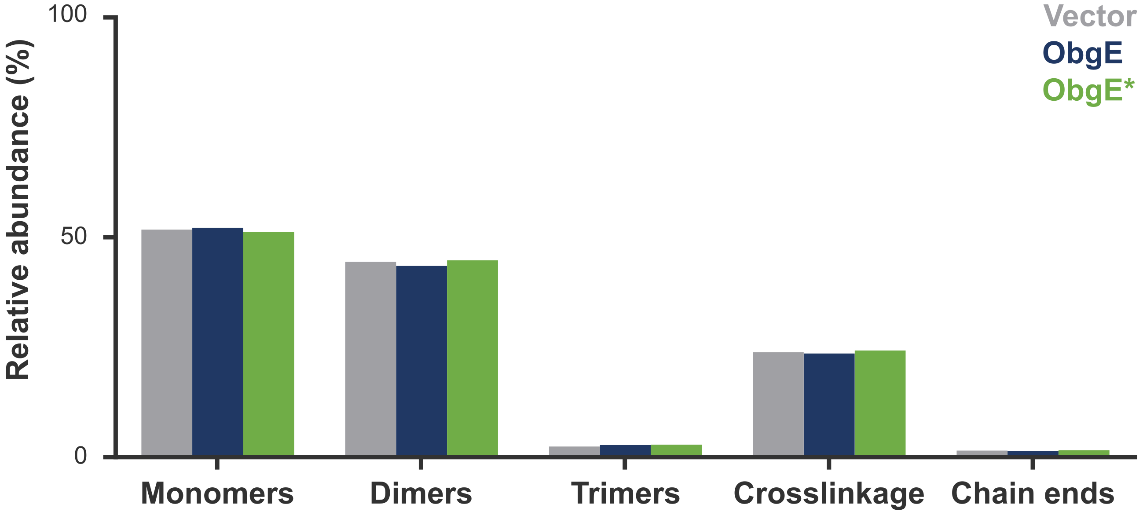


**Figure S1: Peptidoglycan composition is not altered by ObgE*.** The relative abundance of different groups of muropeptides in *E. coli* BW25113 Δ*recA* containing pBAD33Gm, pBAD33Gm-*obgE* or pBAD33Gm-*obgE** is shown. Monomers, uncrosslinked muropeptides; dimers and trimers, cross-linked muropeptides with two and three peptide stems, respectively. The degree of crosslinkage was calculated as published ([Glauner, 1988](#_ENREF_3)), the chain ends are the percentage of anhydro-muropeptides.


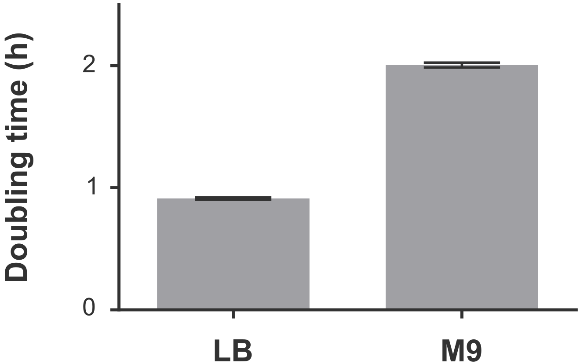


**Figure S2: Doubling time is increased in minimal M9 medium.** Doubling time of *E. coli* pBAD33 in rich LB or minimal M9 medium. Data are represented as mean ± SEM, n = 5.


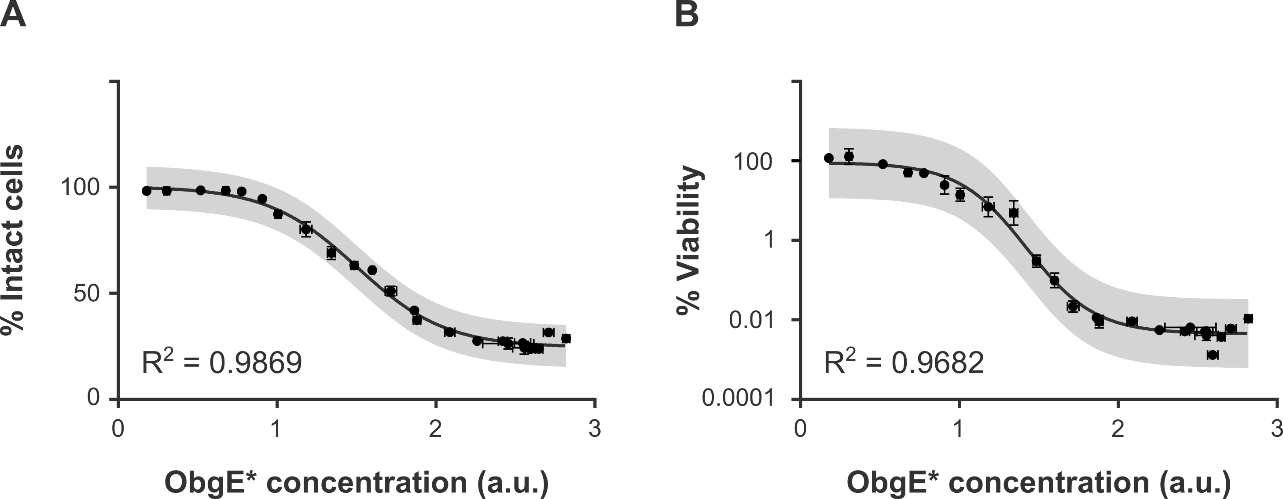


**Figure S3: Construction of correlation curves that predict the amount of lysis and loss of viability based on ObgE* concentration.** A) Measurements of the percentage intact cells of *E. coli* WM2949 pJAT8-*araE* pBAD33-*obgE** at 25 different ObgE* concentrations. *E. coli* WM2949 pJAT8-*araE* allows for a homogeneous induction of the P_BAD_ promotor. B) Measurements of the level of viability of *E. coli* WM2949 pJAT8-*araE* pBAD33-*obgE** in comparison to *E. coli* WM2949 pJAT8-*araE* pBAD33-*obgE* at 25 different expression levels. ObgE* concentration was determined by measuring fluorescence of an ObgE*-Venus fusion by flow cytometry. Data points were fitted to a sigmoidal standard curve. Gray bands around the fitted curves represent 99 % prediction intervals. Data points are represented as mean ± SEM, n = 3.


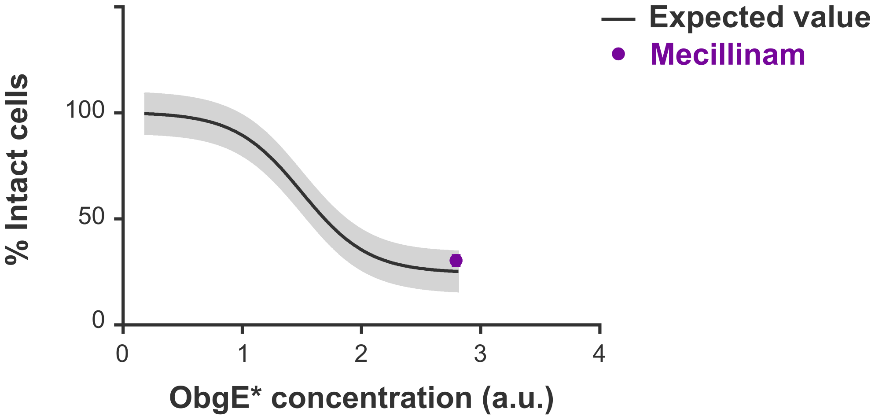


**Figure S4: Cell elongation is not necessary for ObgE*-mediated lysis.** Correlation curve showing the expected fraction of intact cells of *E. coli* pBAD33-*obgE** in function of the intracellular ObgE* concentration. The purple data point represents the percentage intact cells in the presence of mecillinam. ObgE* concentration was determined by measuring fluorescence of an ObgE*-Venus fusion by flow cytometry. Gray bands around the expected value represent 99 % prediction intervals. Data are represented as mean ± SEM, n = 3, error bars are too small to be visible.


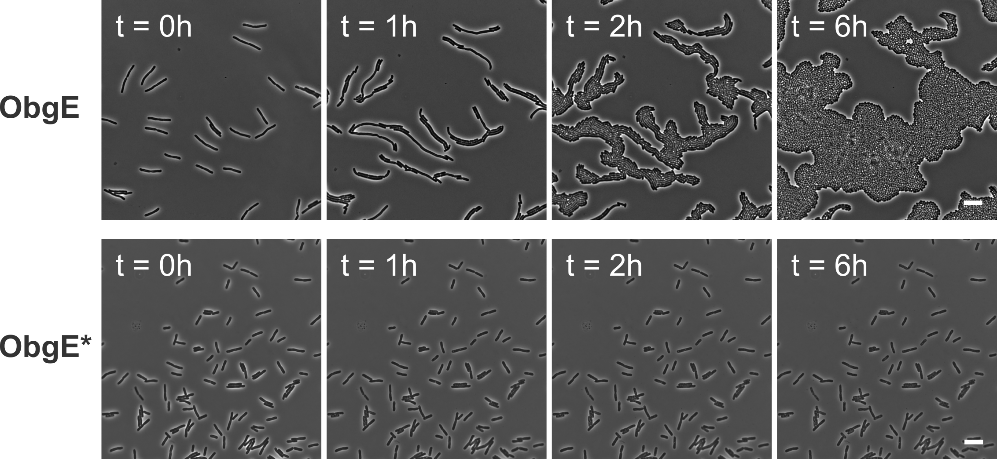


**Figure S5: ObgE* causes irreversible cell cycle arrest independently of exit from stationary phase.** After ObgE or ObgE* was expressed in exponential phase in the presence of aztreonam, cultures were washed and seeded on an agarose pad without the inducer of expression and without aztreonam. Resumption of growth on the pad was monitored by time lapse microscopy. Scale bars, 10 µm.

## Supplementary tables

**Table S1: Minimal inhibitory concentrations (MIC) and Minimal bactericidal concentrations (MBC) in *E. coli* BW25113 (µg/ml).**

|  | MIC | MBC | Chosen conc. |
| --- | --- | --- | --- |
| Aztreonam | 0.16 | 0.32 | 0.2 |
| Cephalexin | 32 | 64-128 | 50 |
| Mecillinam | 0.50 | 1-2 | 0.64 |
|  |  |  |  |

# Supplementary references

Bui, N. K., Gray, J., Schwarz, H., Schumann, P., Blanot, D. & Vollmer, W. (2009). The peptidoglycan sacculus of *Myxococcus xanthus* has unusual structural features and is degraded during glycerol-induced myxospore development. J Bacteriol *191*, 494-505.

Gerits, E., et al. (2017). Repurposing AM404 for the treatment of oral infections by *Porphyromonas gingivalis*. Clin Exp Dent Res. *3*, 69-76.

Glauner, B. (1988). Separation and quantification of muropeptides with high-performance liquid chromatography. Anal Biochem *172*, 451-64.

Guzman, L. M., Belin, D., Carson, M. J. & Beckwith, J. (1995). Tight regulation, modulation, and high-level expression by vectors containing the arabinose P_BAD_ promoter. J Bacteriol *177*, 4121-30.

Khlebnikov, A., Datsenko, K. A., Skaug, T., Wanner, B. L. & Keasling, J. D. (2001). Homogeneous expression of the P_BAD_ promoter in *Escherichia coli* by constitutive expression of the low-affinity high-capacity AraE transporter. Microbiology *147*, 3241-7.

Morgan-Kiss, R. M., Wadler, C. & Cronan, J. E., Jr. (2002). Long-term and homogeneous regulation of the *Escherichia coli araBAD* promoter by use of a lactose transporter of relaxed specificity. Proc Natl Acad Sci U S A *99*, 7373-7.

Quandt, J. & Hynes, M. F. (1993). Versatile suicide vectors which allow direct selection for gene replacement in Gram-negative bacteria. Gene *127*, 15-21.

Zwietering, M. H., Jongenburger, I., Rombouts, F. M. & Van 'T Riet, K. (1990). Modeling of the bacterial growth curve. Appl Environ Microbiol *56*, 1875-81.
